# Anti-inflammatory effect of curcuminoids and their analogs in hyperosmotic human corneal limbus epithelial cells

**DOI:** 10.1186/s12906-024-04448-8

**Published:** 2024-04-23

**Authors:** Ngamjit Kasetsuwan, Usanee Reinprayoon, Lita Uthaithammarat, Amornpun Sereemaspun, Nutchanart Sae-liang, Waraluck Chaichompoo, Apichart Suksamrarn

**Affiliations:** 1 Department of Ophthalmology, Faculty of Medicine, Chulalongkorn University and King Chulalongkorn Memorial Hospital, Bangkok, Thailand; 2 Department of Ophthalmology, Center of Excellence for Cornea and Stem Cell Transplantation, Faculty of Medicine, Chulalongkorn University and King Chulalongkorn Memorial Hospital, Thai Red Cross Society, Bangkok, Thailand; 3 Department of Anatomy, Center of Excellence in Nanomedicine, Faculty of Medicine, Chulalongkorn University, Bangkok, Thailand; 4 Department of Chemistry and Center of Excellence for Innovation in Chemistry, Faculty of Science, Ramkhamhaeng University, Bangkok, Thailand

**Keywords:** Curcuminoids, Curcumin, Anti-inflammatory, Cytokines, Cell viability, Primary human corneal limbal epithelial cells

## Abstract

**Background:**

To assess the efficacy of curcuminoids (curcumin, demethoxycurcumin, bisdemethoxycurcumin [BDC]) and their analogs (tetrahydrocurcumin [THC], tetrahydrodemethoxycurcumin [THDC], tetrahydrobisdemethoxycurcumin) in reducing inflammatory cytokines and their toxicity to primary human corneal limbal epithelial cells, these cells were cultured and exposed to these compounds.

**Methods:**

The PrestoBlue assay assessed cell viability after treatment. Anti-inflammatory effects on hyperosmotic cells were determined using real-time polymerase chain reaction and significance was gauged using one-way analysis of variance and Tukey’s tests, considering p-values < 0.05 as significant.

**Results:**

Curcuminoids and their analogs, at 1, 10, and 100 µM, exhibited no effect on cell viability compared to controls. However, cyclosporin A 1:500 significantly reduced cell viability more than most curcuminoid treatments, except 100 µM curcumin and BDC. All tested curcuminoids and analogs at these concentrations significantly decreased mRNA expression levels of tumor necrosis factor-α, interleukin (IL)-1β, IL-6, IL-17 A, matrix metallopeptidase-9, and intercellular adhesion molecule-1 after 90 mM NaCl stimulation compared to untreated cells. Furthermore, proinflammatory cytokine levels from hyperosmotic cells treated with 1, 10, and 100 µM curcumin, 100 µM BDC, 100 µM THC, 1 and 100 µM THDC mirrored those treated with cyclosporin A 1:500.

**Conclusion:**

The anti-inflammatory efficiency of 1 and 10 µM curcumin, 100 µM THC, 1 and 100 µM THDC was comparable to that of cyclosporin A 1:500 while maintaining cell viability.

**Supplementary Information:**

The online version contains supplementary material available at 10.1186/s12906-024-04448-8.

## Background

Turmeric (*Curcuma longa*) is a plant in the ginger family. Curcuminoids are yellow chemical compounds found in turmeric, comprising curcumin (diferuloylmethane) (77%), demethoxycurcumin (DC) (17%), and bisdemethoxycurcumin (BDC) (3%) [1]. Historically, curcuminoids have demonstrated numerous biological activities at the molecular level, such as anti-inflammatory, antioxidant, proapoptotic, and anti-cancer properties. These effects have been beneficial in many human disease studies, including rheumatoid arthritis, psoriasis, inflammatory bowel disease, irritable bowel syndrome, gastric ulcer, Alzheimer’s disease, and cardiovascular disease. Furthermore, curcuminoids have also shown positive effects in trials related to ophthalmologic diseases such as chronic anterior uveitis [2], age-related macular degeneration [3], diabetic retinopathy [2], central serous chorioretinopathy [2], retinal ischemia [2], and dry eye disease [4], among others.

Tetrahydrocurcumin (THC), a major plasma metabolite of curcumin and a curcuminoid analog, is colorless and more hydrophilic and stable than curcumin due to its non-planar form and benzene rings located at the end of the heptane chain. THC also exhibits a higher antioxidant activity than curcumin and possesses various biological properties, including anti-inflammatory, chemopreventive, antibacterial, antidyslipidemic, antiviral, cytotoxic, antiangiogenic, neurological, antihistamine, immunological, and anti-aging activities [5]. Modifications of curcuminoids’ structural motifs [6], such as THC, tetrahydrodemethoxycurcumin (THDC), and tetrahydrobisdemethoxycurcumin (THBDC), have been introduced to grant these curcuminoid analogs similar biological effects to curcuminoids with enhanced stability and solubility [5].

Dry eye disease (DED) is an ocular surface ailment causing various symptoms such as photophobia, gritty eyes, and visual disturbances. Inflammation is a critical component in the DED cycle [7]. Currently, there are several treatment options for DED, including topical eye drops, oral medications, lid hygiene combined with warm compression, and device-based treatments. Artificial tear drops are typically the initial treatment choice. Topical corticosteroids and cyclosporin eye drops can effectively reduce inflammation in DED [8]. However, topical corticosteroids may lead to secondary glaucoma and cataract formation, whereas topical cyclosporin can cause a burning and stinging sensation during application, paired with a slow onset of action and a higher price [9].

Due to their biological activity, curcuminoids and their analogs have potential as treatments for numerous diseases in the future, including DED. In this study, we aim to examine the capability of curcuminoids (curcumin, DC, and BDC) and curcuminoid analogs (THC, THDC, and THBDC) in diminishing inflammatory cytokines and to observe the toxicity of these substances on primary human corneal limbal epithelial cells. This research could pave the way for innovative eye drops as a treatment for DED.

## Methods

### Curcuminoids and Curcuminoid Analogs

**Fig. 1:**
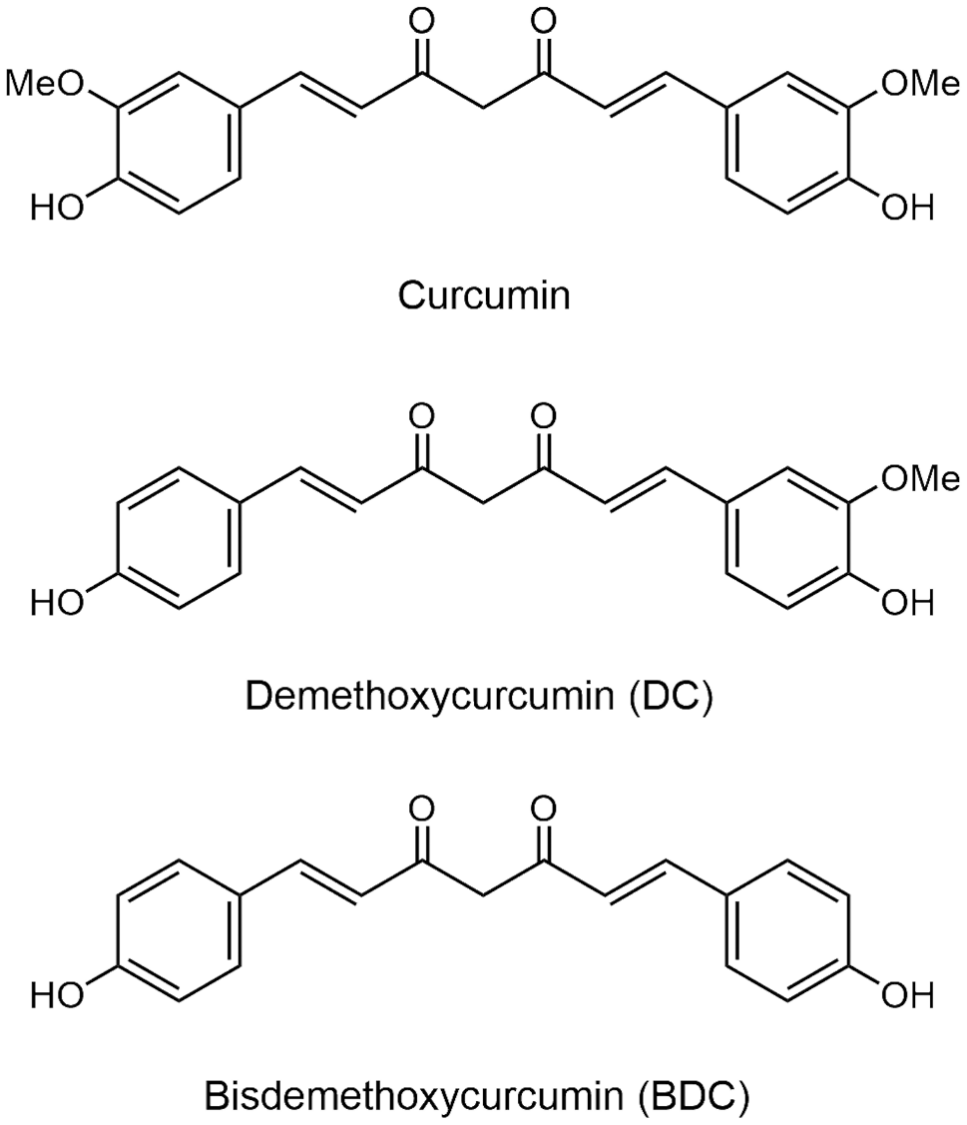
Curcumin (diferuloylmethane), demethoxycurcumin, and bisdemethoxycurcumin

**Fig. 2:**
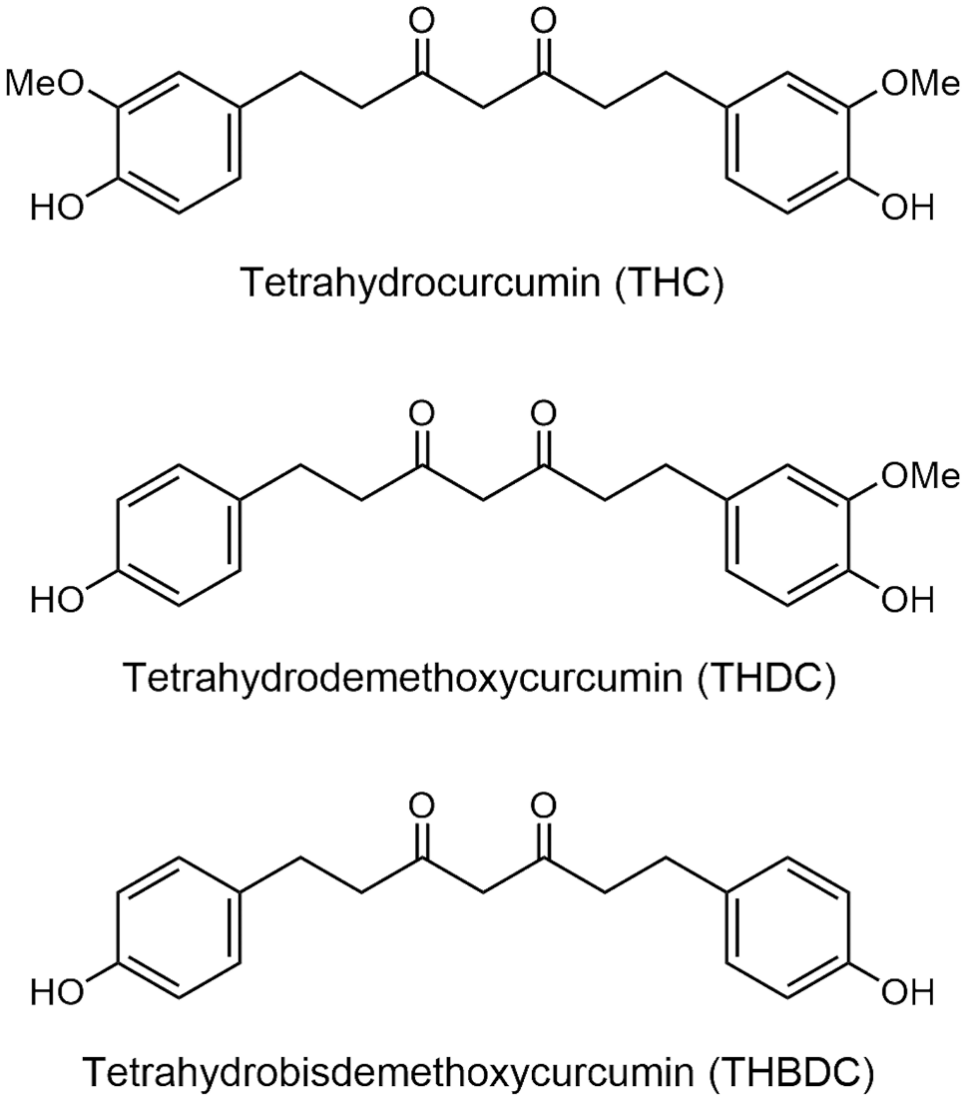
Tetrahydrocurcumin, tetrahydrodemethoxycurcumin, and tetrahydrobisdemethoxycurcumin

The parent curcuminoids (curcumin, DC, and BDC), illustrated in Fig. 1, were isolated from the rhizomes of *Curcuma longa* L. (Zingiberaceae) [10]. The plant materials were purchased from Chatuchak local market, Bangkok, Thailand in January 2014 and were identified by Assoc. Prof. Nopporn Dumrongsiri, Department of Biology, Faculty of Science, Ramkhamhaeng University. A voucher specimen (Apichart Suksamrarn, No. 073) was deposited at the Department of Chemistry, Faculty of Science, Ramkhamhaeng University. These curcuminoids were chemically modified to produce the corresponding tetrahydro analogs THC, THDC, and THBDC, as depicted in Fig. 2, utilizing the method previously described [11]. Starting with curcumin, catalytic hydrogenation, employing palladium on charcoal as a catalyst, yielded 76% THC and 9% hexahydrocurcumin. The spectroscopic data for these isolated analogs aligned with previously reported values [12, 13]. For DC, the tetrahydro analog THDC and hexahydrodemethoxycurcumin were produced in yields of 70% and 8%, respectively. The spectroscopic data of these synthesized analogs matched previously reported values [14]. From BDC, the tetrahydro analog THDC and hexahydrobisdemethoxycurcumin were obtained in 71% and 8% yields, respectively. Similarly, the spectroscopic data for these synthesized analogs were consistent with the reported values [14]. The purity of both the isolated curcuminoids (curcumin, DC, and BDC) and the synthesized analogs (THC, THDC, and THBDC) was ascertained via thin-layer chromatography (TLC). This was conducted using Merck’s precoated silica gel 60 F254 plates, and dichloromethane–methanol and n-hexane–ethyl acetate were used as the developing solvent systems. Spots on the TLC were detected under UV light and subsequently by spraying with an anisaldehyde–sulfuric acid reagent and heating. The compounds’ purity was further validated by nuclear magnetic resonance spectroscopy. All the compounds were dissolved in dimethyl sulfoxide to prepare a 30 mM stock solution and then added to the medium in varying concentrations. The curcuminoid solution exhibited a yellow hue, whereas the solution derived from curcuminoid analogs was colorless.

### Feeder cell preparation

Murine 3T3 fibroblast feeder cells were cultured in Dulbecco’s Modified Eagle’s Medium (DMEM) (HyClone, Logan, UT, USA) supplemented with 10% fetal bovine serum (Gibco, Grand Island, NY, USA), 1% GlutaMax (Gibco, Grand Island, NY, USA), and 1% antibiotic-antimycotic (Gibco, Grand Island, NY, USA). They were incubated at 37 °C in an atmosphere containing 5% CO_2_. Once the cells reached approximately 80–90% confluence, they were treated with media containing 10 µg/mL mitomycin C (Sigma, St. Louis, MO) at 37 °C for 2 h. Subsequently, the media was discarded, and the cells were washed with 1X phosphate-buffered saline (PBS). These cells were then trypsinized and plated in 60-mm dishes at a density of 1 × 10^6^ cells, followed by incubation at 37 °C under 5% CO_2_. The feeder cells were used 4–24 h post-seeding. The 3T3 feeder cells were used as a feeder layer for the growth and expansion of limbal epithelial cells.

### 3T3-J2-conditioned medium preparation

3T3-J2 fibroblast feeder cells were cultured in DMEM with 10% bovine calf serum (Gibco, Grand Island, NY, USA). This was done at 37 °C in a 10% CO_2_-humidified incubator. The 3T3-J2-conditioned medium was prepared by inactivating the 3T3-J2 fibroblast feeder cells using 10 µg/mL of mitomycin C (Sigma, St. Louis, MO) at 37 °C for 2 h. Then, these cells were rinsed twice with sterile PBS and subsequently treated with epithelial cell culture medium lacking growth factors for 24 h. The 3T3-J2-conditioned medium was then obtained by centrifugation, filtered through a 0.45 µM filter, and stored at 4 °C. The medium was used within 7 days after preparation.

### Primary human corneal limbal epithelial cell culture

Primary human corneal limbal epithelial cells, which were derived from human donor cornea, were sourced from the Excellence Center for Stem Cell and Cell Therapy, Faculty of Medicine, Chulalongkorn University, Thailand. The marker of these cells were p63 alpha. These cells were co-cultured with dishes containing 3T3 feeder cells in DMEM: Nutrient Mixture F-12 (DMEM/F12, 1:1). The mixture was supplemented with 10% fetal bovine serum (Gibco, Grand Island, NY, USA), 2.5 µg/mL NaHCO_3_ (HyClone, Logan, UT, USA), 0.5 µg/mL hydrocortisone (Sigma, St. Louis, MO), 1% GlutaMax (Gibco, Grand Island, NY, USA), 1% antibiotic-antimycotic (Gibco, Grand Island, NY, USA), 5 mg/mL human insulin, 20 ng/mL recombinant human epidermal growth factor protein (Bio-Techne, USA), and 0.5 µM ALK5 inhibitor (Miltenyi Biotec, Germany). The culture was maintained at 37 °C in a 5% CO_2_-humidified incubator for 14 days to facilitate the growth of limbal epithelial cell colonies. After 14 days, the 3T3 feeder layers were detached using 0.25% trypsin/ethylenediaminetetraacetic acid (EDTA) (Gibco, Grand Island, NY, USA) at 37 °C for 1 min. The limbal epithelial cell colonies were then dissociated with 0.25% trypsin/EDTA at 37 °C for 5 min to yield single limbal epithelial cells. Subsequently, the cells from the 2nd passage were seeded onto dishes coated with 10 µg/mL collagen type I (Bio-Techne, USA) at a density of 5 × 10_5_ cells and bathed in 3T3-J2-conditioned medium for further experiments.

### Cell viability assay

The cytotoxicity of curcuminoids and their analogs on primary human corneal limbal epithelial cells was assessed using PrestoBlue™ cell viability reagent (Invitrogen, Waltham, MA, USA). Cells were plated in 96-well plates at a density of 5 × 10^3^ cells/well in serum-free media and were incubated at 37 °C with 5% CO_2_ for 24 h. Curcuminoids (curcumin, DC, and BDC) and curcuminoid analogs (THC, THDC, and THBDC) were introduced at final concentrations of 0, 1, 10, and 100 µM. Cyclosporin A at 0.05% was diluted 1:500 in DMEM/F12. The cells, in the presence of curcuminoids, curcuminoid analogs, and cyclosporin A, were further incubated for 24 h at 37 °C with 5% CO_2_. Subsequently, they were treated with 10 µL of PrestoBlue™ reagent for 30 min. Cell viability was quantified by measuring fluorescence intensity using a microplate reader at wavelengths of 560 nm and 590 nm (Thermo Scientific, Waltham, MA, USA). The control group of primary human corneal limbal epithelial cells (untreated with any reagent) was set as the benchmark for 100% viability.

### Hyperosmoticity assay

Cells were seeded into 6-well plates at a density of 5 × 10^5^ cells/well and incubated for 24 h. Following this, cells were exposed to 90 mM sodium chloride (NaCl) to induce a hyperosmoticity of 450 mOsM for 6 h. Post NaCl exposure, cells were treated with either cyclosporin A at a dilution of 1:500 or with curcuminoids and their analogs at final concentrations of 0, 1, 10, and 100 µM. Subsequently, they were incubated for an additional 24 h at 37 °C in an atmosphere containing 5% CO_2_.

### RNA extraction and real-time PCR

After the 24-h incubation, cells were harvested by centrifugation for RNA extraction. Total RNA was isolated using the TRIZol^®^ reagent (Invitrogen, Waltham, MA, USA). The RNA concentration and quality were ascertained using a NanoDrop spectrophotometer (Thermo Fisher Scientific, Waltham, MA, USA) with measurements taken at 260 nm. The cDNA synthesis was achieved using the RevertAid First Strand cDNA synthesis kit (Thermo Fisher Scientific, Waltham, MA, USA), adhering to the manufacturer’s guidelines. Quantitative reverse transcription PCR was executed using the Power Up™ SYBR™ Green Master Mix in accordance with the provided instructions. The PCR amplification was carried out on the QuantStudio™ 6 Flex Real-time PCR system (Thermo Fisher Scientific, Waltham, MA, USA) under the stipulated conditions: 50 °C for 2 min, 95 °C for 2 min, followed by 40 cycles of 95 °C for 5 s and 60 °C for 30 s. The expression levels of target genes were normalized against the endogenous glyceraldehyde 3-phosphate dehydrogenase (GAPDH) levels and then compared to a normalized calibrator. The primers used in this study, which include tumor necrosis factor (TNF)-α, interleukin (IL)-1β, IL-6, IL-17 A, matrix metallopeptidase-9 (MMP-9), intercellular adhesion molecule-1 (ICAM-1), and GAPDH, are itemized in Table S1.

### Statistical analysis

Statistical analysis was conducted using GraphPad Prism version 9. Differences in means were assessed for statistical significance using one-way analysis of variance (ANOVA), followed by Tukey’s multiple comparison tests. *P*-values < 0.05 were deemed significant.

## Results

### Cell viability assay

**Fig. 3:**
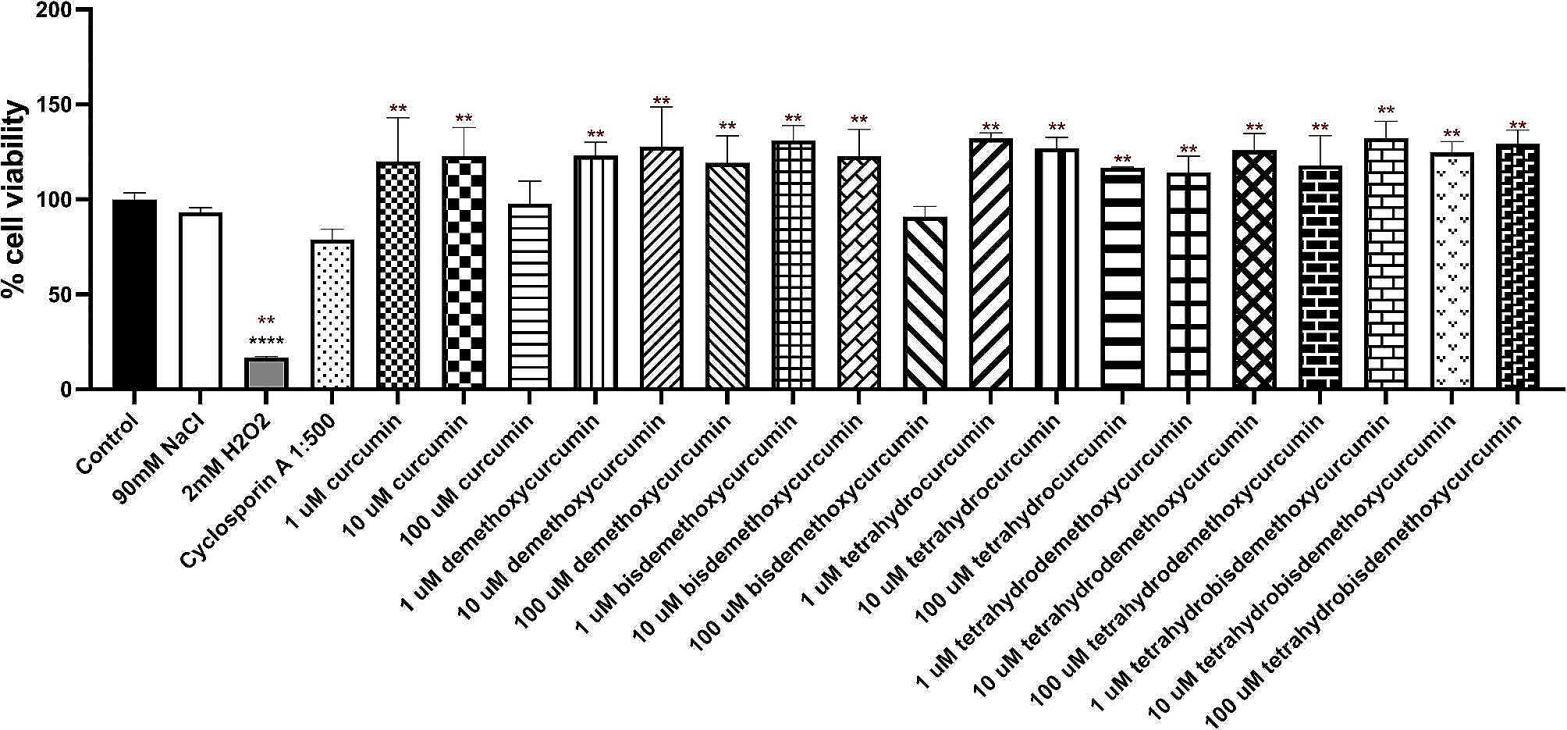
Effect of curcuminoids and curcuminoid analogs on primary human corneal limbal epithelial cells at concentrations of 1, 10, and 100 µM over 24 h. Cell viability kicker viability was determined using PrestoBlue*™* cell viability reagent. **P < 0.0001, compared with the control group; and *P < 0.05, compared with the cyclosporin A 1:500 group

The viability of primary human corneal limbal epithelial cells, when treated with curcuminoids and their analogs at varying concentrations (1, 10, and 100 µM) for 24 h, showed no significant difference in comparison to the control group (*P* > 0.05) (Fig. 3). As a result, curcuminoids and curcuminoid analogs at concentrations of 100 µM or less were employed in subsequent experiments. Additionally, certain curcuminoid analogs displayed significantly higher cell viability in primary human corneal limbal epithelial cells compared to those treated with cyclosporin A at a 1:500 ratio, as detailed in Table 1. The mean cell viability, complete with standard deviation and a 95% confidence interval for all agents, is illustrated in Table S2.

**Table 1:** Cell viability of cyclosporin A 0.05% 1:500 compared with each analog

Tukey’s multiple comparisons test	Mean difference	95% CI of difference	Adjusted P-value
2 mM H_2_O_2_ vs. cyclosporin A 1:500	−62.17	−95.46 to − 28.87	< 0.0001
cyclosporin A 1:500 vs. 1 μm curcumin	−41.09	−74.38 to − 7.79	0.0040
cyclosporin A 1:500 vs. 10 μm curcumin	−43.91	−77.20 to − 10.62	0.0015
cyclosporin A 1:500 vs. 100 μm curcumin	−18.87	−52.16 to 14.43	0.8378
cyclosporin A 1:500 vs. 1 μm DC	−44.20	−77.50 to − 10.91	0.0013
cyclosporin A 1:500 vs. 10 μm DC	−48.85	−82.15 to − 15.56	0.0002
cyclosporin A 1:500 vs. 100 μm DC	−40.33	−73.62 to − 7.04	0.0052
cyclosporin A 1:500 vs. 1 μm BDC	−52.03	−85.32 to − 18.73	< 0.0001
cyclosporin A 1:500 vs. 10 μm BDC	−43.93	−77.22 to − 10.63	0.0015
cyclosporin A 1:500 vs. 100 μm BDC	−11.98	−45.28 to 21.31	0.9983
cyclosporin A 1:500 vs. 1 μm THC	−53.21	−86.51 to − 19.92	< 0.0001
cyclosporin A 1:500 vs. 10 μm THC	−48.04	−81.33 to − 14.75	0.0003
cyclosporin A 1:500 vs. 100 μm THC	−37.84	−71.13 to − 4.54	0.0120
cyclosporin A 1:500 vs. 1 μm THDC	−35.35	−68.64 to − 2.05	0.0268
cyclosporin A 1:500 vs. 10 μm THDC	−47.01	−80.30 to − 13.71	0.0005
cyclosporin A 1:500 vs. 100 μm THDC	−39.00	−72.30 to − 5.71	0.0081
cyclosporin A 1:500 vs. 1 μm THBDC	−53.32	−86.61 to − 20.02	< 0.0001
cyclosporin A 1:500 vs. 10 μm THBDC	−45.86	−79.16 to − 12.57	0.0007
cyclosporin A 1:500 vs. 100 μm THBDC	−50.61	−83.90 to − 17.31	0.0001

CI = confidence interval, DC = demethoxycurcumin, BDC = bisdemethoxycurcumin, THC = tetrahydrocurcumin, THDC = tetrahydrodemethoxycurcumin, and THBDC = tetrahydrobisdemethoxycurcumin

### Proinflammatory cytokine mRNA expression levels

**Fig. 4:**
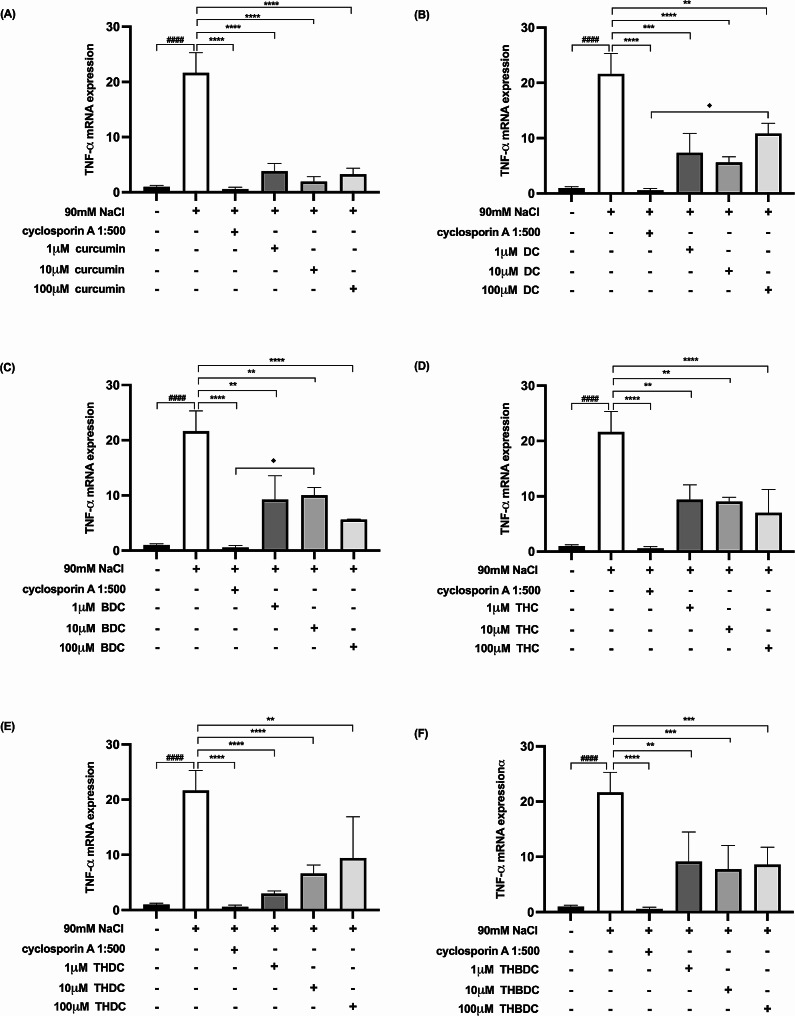
Effect of curcuminoids and curcuminoid analogs on TNF-α mRNA expressions in hyperosmotic-induced human corneal limbal epithelial cells. After 90mM NaCl exposure for 6 h, TNF-α mRNA expression levels were inhibited by (**A**) curcumin, (**B**) demethoxycurcumin (DC), (**C**) bisdemethoxycurcumin (BDC), (**D**) tetrahydrocurcumin (THC), (**E**) tetrahydrodemethoxycurcumin (THDC), and (**F**) tetrahydrobisdemethoxycurcumin (THBDC). mRNA expression levels were assessed using quantitative RT-PCR. ^*####*^*P* < *0.0001* compared to control. ^**^P < *0.01*, ^***^P < *0.001*, ^****^P < *0.0001* compared to NaCl alone. ^♦^*P* < *0.05* compared to NaCl + cyclosporin A

**Fig. 5:**
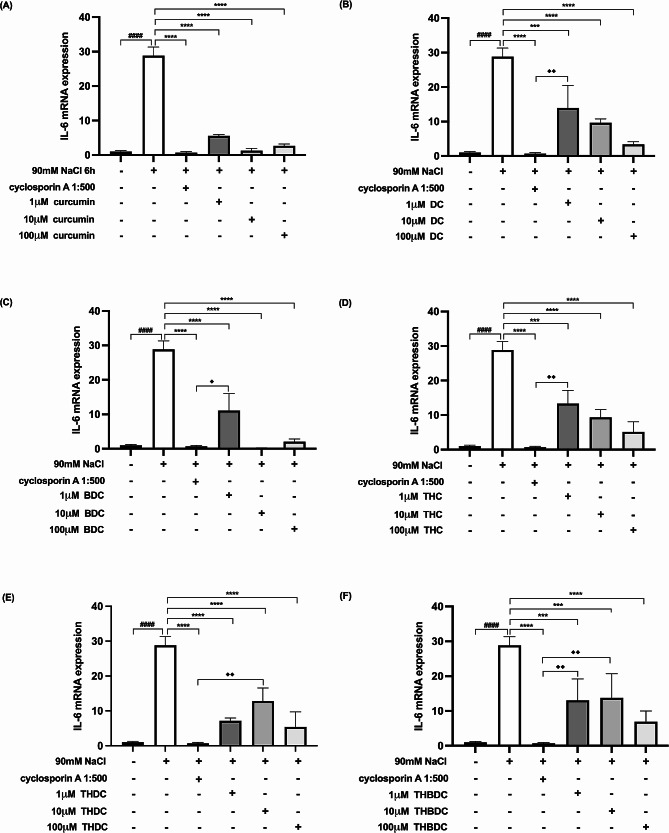
Effect of curcuminoids and curcuminoid analogs on IL-6 mRNA expressions in hyperosmotic-induced human corneal limbal epithelial cells. After 90mM NaCl exposure for 6 h, IL-6 mRNA expression levels were inhibited by (**A**) curcumin, (**B**) demethoxycurcumin (DC), (**C**) bisdemethoxycurcumin (BDC), (**D**) tetrahydrocurcumin (THC), (**E**) tetrahydrodemethoxycurcumin (THDC), and (**F**) tetrahydrobisdemethoxycurcumin (THBDC). mRNA expression levels were assessed using quantitative RT-PCR. ^*####*^*P* < *0.0001* compared to control. ^***^P < *0.001*, ^****^P < *0.0001* compared to NaCl alone. ^♦^*P* < *0.05*, ^♦♦^*P* < *0.01* compared to NaCl + cyclosporin A

**Fig. 6:**
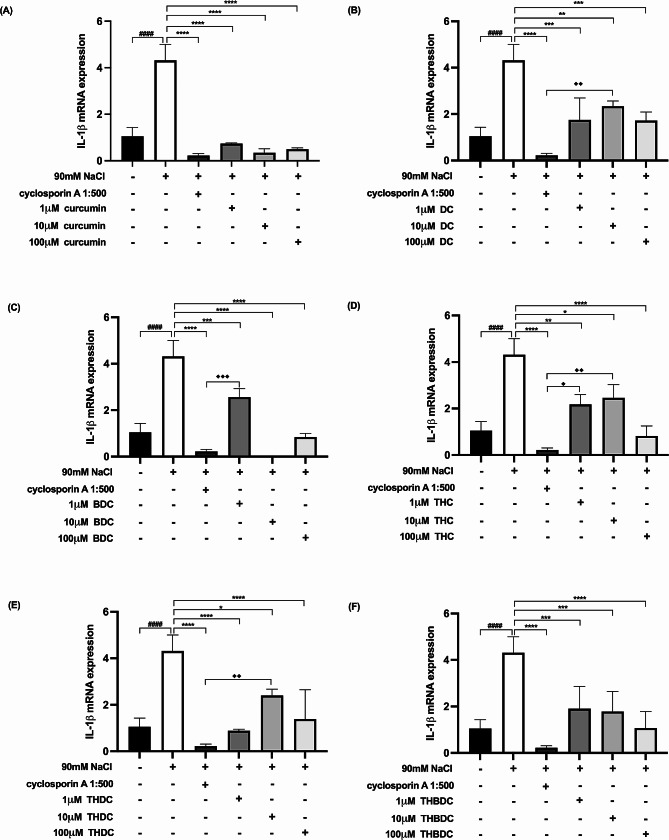
Effect of curcuminoids and curcuminoid analogs on IL-1β mRNA expressions in hyperosmotic-induced human corneal limbal epithelial cells. After 90mM NaCl exposure for 6 h, IL-1β mRNA expression levels were inhibited by (**A**) curcumin, (**B**) demethoxycurcumin (DC), (**C**) bisdemethoxycurcumin (BDC), (**D**) tetrahydrocurcumin (THC), (**E**) tetrahydrodemethoxycurcumin (THDC), and (**F**)tetrahydrobisdemethoxycurcumin (THBDC). mRNA expression levels were assessed using quantitative RT-PCR. ^*####*^*P* < *0.0001* compared to control. ^*^P < *0.05*, ^**^P < *0.01*, ^***^P < *0.001*, ^****^P < *0.0001* compared to NaCl alone.  ^♦^*P* < *0.05*, ^♦♦^*P* < *0.01 compared to NaCl + cyclosporin A*

**Fig. 7:**
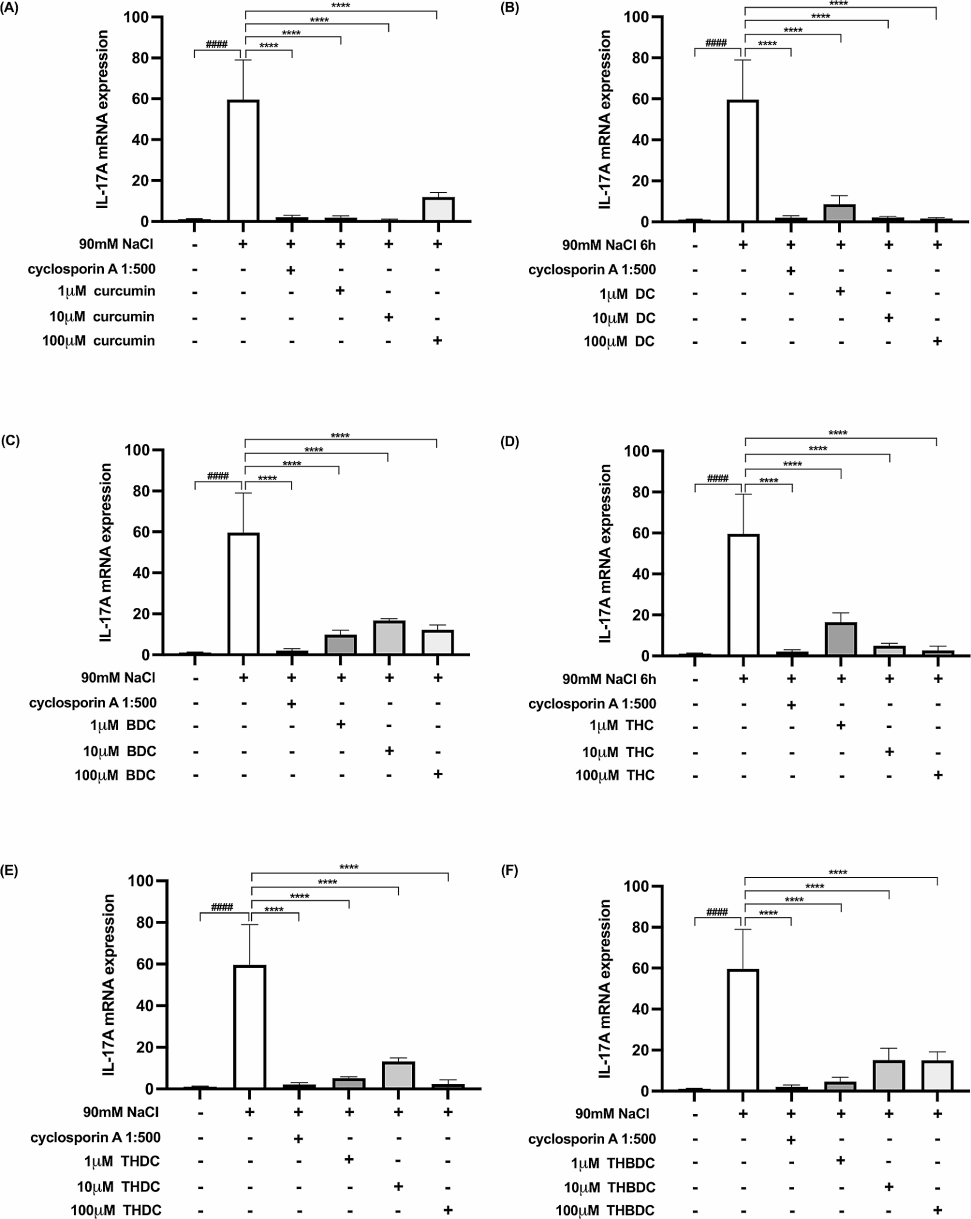
Effect of curcuminoids and curcuminoid analogs on IL-17 A mRNA expressions in hyperosmotic-induced human corneal limbal epithelial cells. After 90mM NaCl exposure for 6 h, IL-17 A mRNA expression levels were inhibited by (**A**) curcumin, (**B**) demethoxycurcumin (DC), (**C**) bisdemethoxycurcumin (BDC), (**D**) tetrahydrocurcumin (THC), (**E**) tetrahydrodemethoxycurcumin (THDC), and (**F**) tetrahydrobisdemethoxycurcumin (THBDC). mRNA expression levels were assessed using quantitative RT-PCR. ^*####*^*P* < *0.0001* compared to control. ^****^P < *0.0001* compared to NaCl alone

**Fig. 8:**
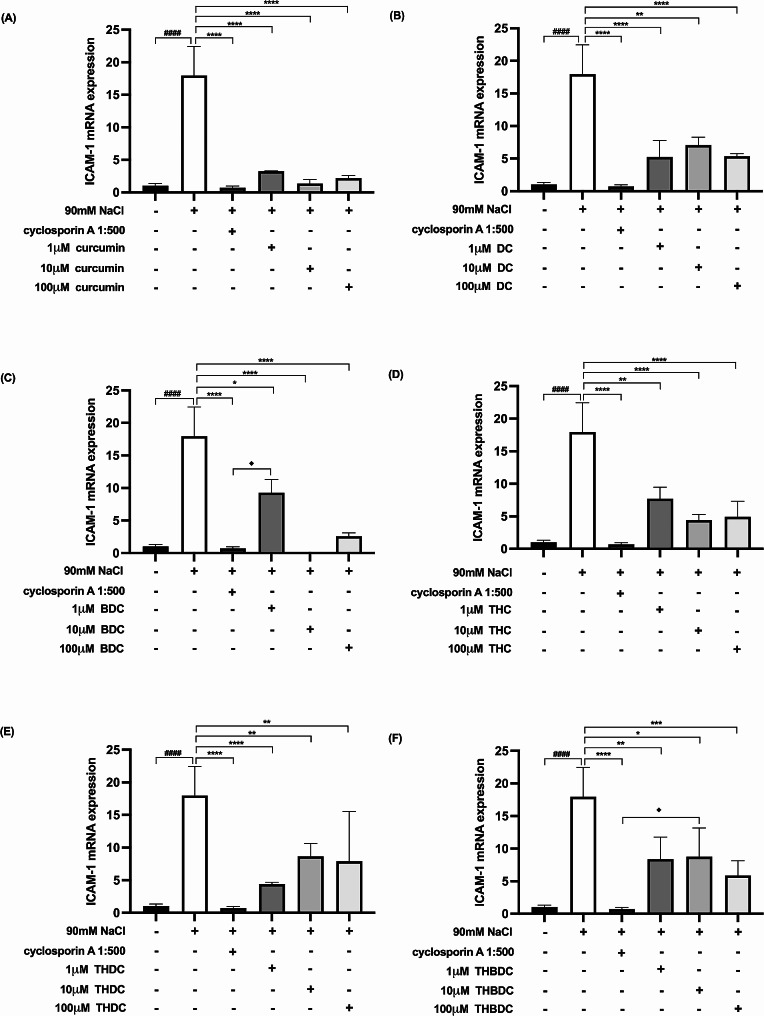
Effect of curcuminoids and curcuminoid analogs on ICAM-1 mRNA expressions in hyperosmotic-induced human corneal limbal epithelial cells. After 90mM NaCl exposure for 6 h, ICAM-1 mRNA expression levels were inhibited by (**A**) curcumin, (**B**) demethoxycurcumin (DC), (**C**) bisdemethoxycurcumin (BDC), (**D**) tetrahydrocurcumin (THC), (**E**) tetrahydrodemethoxycurcumin (THDC), and (**F**) tetrahydrobisdemethoxycurcumin (THBDC). mRNA expression levels were assessed using quantitative RT-PCR. ^*####*^*P* < *0.0001* compared to control. ^*^P < *0.05*, ^**^P < *0.01*, ^***^P < *0.001*, ^****^P < *0.0001* compared to NaCl alone. ^♦^*P* < *0.05* compared to NaCl + cyclosporin A

**Fig. 9:**
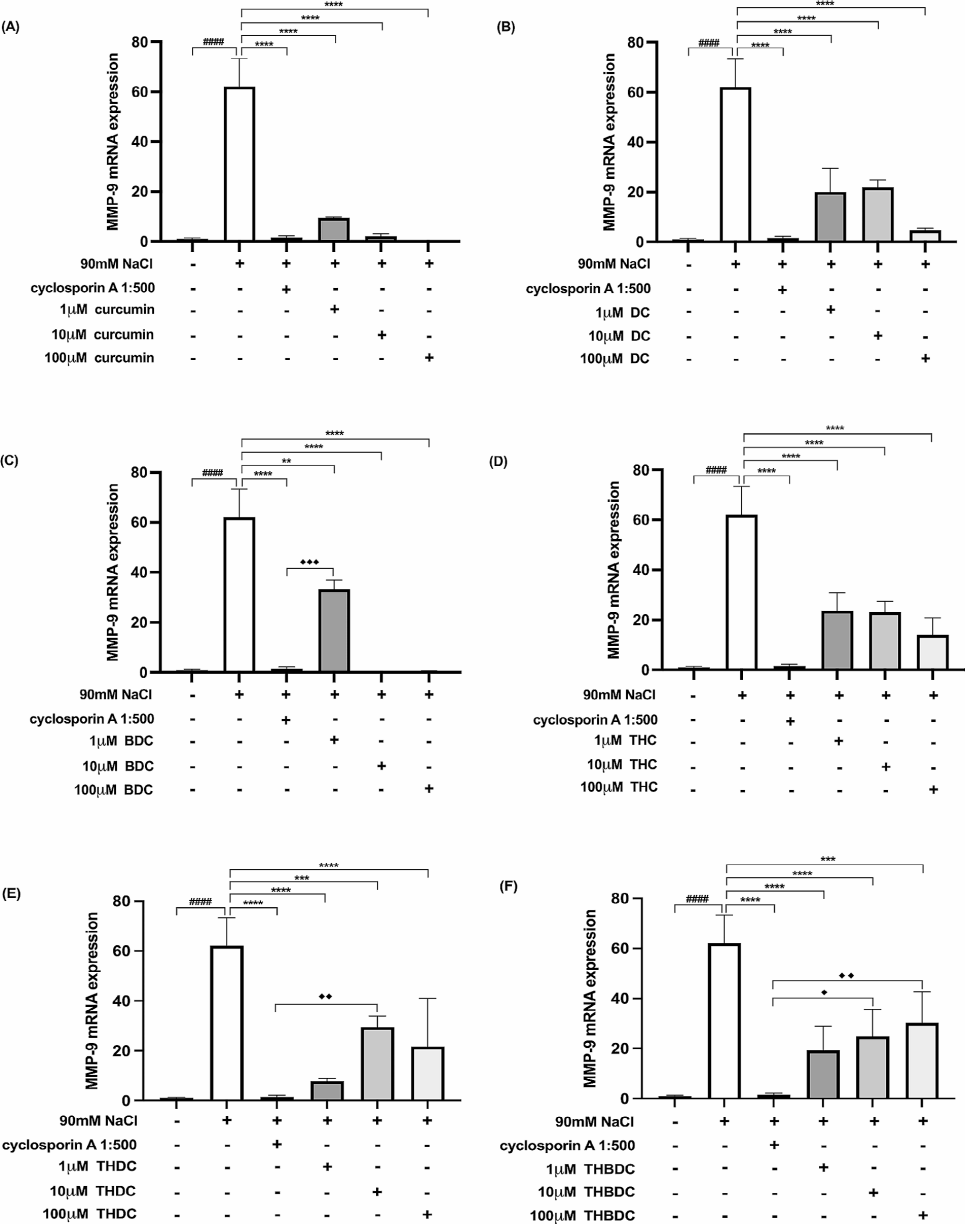
Effect of curcuminoids and curcuminoid analogs on MMP-9 mRNA expressions in hyperosmotic-induced human corneal limbal epithelial cells. After 90mM NaCl exposure for 6 h, ICAM-1 mRNA expression levels were inhibited by (**A**) curcumin, (**B**) demethoxycurcumin (DC), (**C**) bisdemethoxycurcumin (BDC), (**D**) tetrahydrocurcumin (THC), (**E**) tetrahydrodemethoxycurcumin (THDC), and (**F**) tetrahydrobisdemethoxycurcumin (THBDC). mRNA expression levels were assessed using quantitative RT-PCR. ^*####*^*P* < *0.0001* compared to control. ^**^P < *0.01*, ^***^P < *0.001*, ^****^P < *0.0001* compared to NaCl alone. ^♦^*P* < *0.05*, ^♦♦^*P* < *0.01*, ^♦♦♦^*P* < *0.001* compared to NaCl + cyclosporin A

Upon 6-h exposure to 450 mOsM hyperosmoticity (90 mM NaCl), the mRNA expression levels of proinflammatory cytokines surged in hyperosmotic-induced cells. A statistically significant difference (*P* < 0.0001) was observed in mRNA expression levels of all the proinflammatory cytokines (TNF-α, IL-1β, IL-6, IL-17 A, MMP-9, and ICAM-1) between cells treated with and without 90 mM NaCl (Figs. 4, 5, 6, 7, 8 and 9).

Following the 6-h exposure to 90 mM NaCl, the cells were subsequently treated with curcuminoids and their analogs at varying concentrations (1, 10, and 100 µM) over 24 h. The data revealed that both curcuminoids and their analogs suppressed the mRNA expression levels of proinflammatory cytokines (TNF-α, IL-1β, IL-6, IL-17 A, MMP-9, and ICAM-1) instigated by hyperosmoticity, in comparison to the hyperosmotic-induced cells (Figs. 4, 5, 6, 7, 8 and 9).

Furthermore, the concentrations of all proinflammatory cytokines treated with 1 µM curcumin, 10 µM curcumin, 100 µM curcumin, 100 µM BDC, 100 µM THC, 1 µM THDC, and 100 µM THDC were not significantly different from those treated with cyclosporin A at a 1:500 ratio. However, certain proinflammatory cytokine levels from cells treated with 1µM DC, 10 µM DC, 100 µM DC, 1 µM BDC, 10 µM BDC, 1µM THC, 10 µM THC, 10 µM THDC, 1µM THBDC, 10 µM THBDC, and 100 µM THBDC were significantly different compared to levels treated by cyclosporin A at a 1:500 ratio (Figs. 4, 5, 6, 7, 8 and 9). The average mRNA expression level of proinflammatory cytokines in each group is presented in Tables S3–S8.

## Discussion

In this study, curcumin, DC, BDC, THC, THDC, and THBDC at concentrations of 1, 10, and 100 µM demonstrated no influence on cell viability. All curcuminoids and their analogs at these concentrations significantly reduced TNF-α, IL-1β, IL-6, IL-17 A, MMP-9, and ICAM-1 mRNA expression in primary human corneal limbal epithelial cells when stimulated by 90 mM NaCl. This was in comparison to the cytokine mRNA expressions of cells stimulated by 90 mM NaCl without the application of any curcuminoid or its analog. Furthermore, the levels of all proinflammatory cytokines from hyperosmotic cells treated with various concentrations of curcuminoids and their analogs were insignificantly different from those treated with cyclosporin A at a 1:500 ratio.

In previous studies, curcumin and THC, at concentrations ranging from 0.01 to 100 µg/mL, exhibited over 70% cell viability, which decreased with increasing concentrations [5]. Conversely, Chen et al. reported that curcumin concentrations below 30 µM had no effect on cell viability. However, a concentration of 50 µM significantly diminished cell viability [15]. In our study, all curcuminoids and their analogs at 1, 10, and 100 µM retained a cell viability of over 90%. In contrast, cyclosporin A at 1:500 had a lower cell viability (78.97%) than most of the curcuminoids and their analogs, with the exception of 100 µM curcumin and 100 µM BDC. These results confirm that curcuminoid analogs, up to 100 µM, are safer for primary human corneal limbal epithelial cells than cyclosporin A at 1:500.

Stimulating primary human corneal limbal epithelial cells in a hyperosmolarity environment (achieved by adding NaCl) is known to trigger the release of numerous inflammatory cytokines [15]. Our study determined that 90 mM NaCl places these cells in an inflammatory state, resulting in elevated expressions of TNF-α, IL-1β, IL-6, IL-17 A, MMP-9, and ICAM-1 mRNA compared to cells not subjected to a hyperosmolarity environment.

Inflammation plays a pivotal role in the perpetuating cycle of DED. TNF-α, IL-1β, IL-17, and MMP-9 are inflammatory cytokines stimulated by tear hyperosmolarity. These cytokines attract more inflammatory cells to the ocular surface, causing damage to the surface epithelial and goblet cells. This damage exacerbates tear hyperosmolarity, initiating the DED cycle. Both adaptive and innate defense mechanisms are implicated in DED pathology. The innate defense mechanism involves stimulation of pattern recognition receptors, which is associated with increased IL-1, TNF-α, and IL-6 on the ocular surface [16]. A meta-analysis confirmed the presence of IL-17 A, IL-6, IL-1β, and TNF-α in the tears of patients with DED [17]. ICAM-1, an adhesion molecule, binds to inflammatory cells expressing integrin leukocyte functional antigen 1 (LFA-1). This binding is essential for recruiting inflammatory cells to the ocular surface in the DED cycle [16]. To disrupt this cycle, reducing inflammatory cytokines and ICAM-1 has proven effective in treatments such as topical corticosteroids, cyclosporin, and LFA-1 antagonists. However, topical corticosteroids come with side effects, including ocular hypertension, cataracts, and opportunistic infections [8]. Topical cyclosporin can cause ocular pain, redness, and eyelid swelling [18]. Additionally, topical LFA-1 may lead to site irritation, reactions, and dysgeusia [19]. Consequently, there is an ongoing search for novel topical drugs.

The anti-inflammatory activity of curcumin and THC has been established both in vitro and in vivo in numerous studies [5, 15, 20–26]. Curcumin was reported to reduce TNF-α, IL-1β, and MMP-9 in cultured rat corneal epithelial cells [20]. Furthermore, a study that examined the anti-inflammatory action of curcumin in cultured human corneal epithelial cells found that 5 µM curcumin could suppress IL-1β, IL-6, and TNF-α proteins [15]. THC has been shown to decrease TNF-α and IL-1β in mouse splenocytes compared to curcumin [5]. Moreover, THC significantly reduced IL-17 A in allergic asthmatic mice, whereas curcumin did not show a similar effect [23]. THC also reduced TNF-α, IL-1β, and IL-6 in mice [21], IL-1β and MMP-9 in astrocytes and the hypoxic cerebrum of mice [24], IL-1β, IL-6, and TNF-α in the articular cartilage of estrogen-deficient rats [25], and TNF-α in high-fat diet obese mice [26].

According to our study, all concentrations of yellow curcuminoid (curcumin, DC, and BDC) significantly reduced the expression of TNF-α, IL-1β, IL-6, IL-17 A, MMP-9, and ICAM-1 mRNA under hyperosmotic conditions compared to untreated conditions. All curcuminoid analogs, including THC, THDC, and THBDC, which are colorless, also markedly suppressed TNF-α, IL-1β, IL-6, IL-17 A, MMP-9, and ICAM-1 mRNA expression. Additionally, 1 µM curcumin, 10 µM curcumin, 100 µM curcumin, 100 µM BDC, 100 µM THC, 1 µM THDC, and 100 µM THDC reduced all inflammatory cytokine and ICAM-1 expression levels, which were not significantly different from those inhibited by cyclosporin A 1:500. This study is the first to report the anti-inflammatory effects of DC, BDC, THC, THDC, and THBDC in cultured human corneal limbal epithelial cells compared to the anti-inflammatory effects of cyclosporin A 1:500, an approved treatment for DED.

However, curcuminoids have a yellow color, exhibit low solubility, permeability, and absorption, and are rapidly metabolized with swift elimination from the body [27]. Preparing curcuminoid substances in liquid or eye drop form is challenging. Several formulation-enhancing drug delivery systems, such as nanoparticles, liposomes, micelles, and phospholipid complexes [27, 28], have been employed to maximize the benefits of curcuminoids in medicine. Another approach to addressing the pharmacokinetic challenges of curcuminoids involves using analogs [6], such as THC, THDC, THBDC, dihydrocurcumin, hexahydrocurcumin, octahydrocurcumin, hexahydrodemethoxycurcumin, octahydrodemethoxycurcumin, hexahydrobisdemethoxycurcumin, and octahydrobisdemethoxycurcumin, through structural motif modifications [5]. Compared to curcumin, THC is more stable, hydrophilic, soluble at physiological pH, possesses a longer half-life in plasma at 37 °C, and is more absorbable in the intestines with sustained stability in plasma [29, 30]. In this study, aside from curcumin and BDC, which are yellow, 100 µM THC and 1 and 100 µM THDC, which are colorless, effectively suppressed inflammatory mRNA with higher cell viability than cyclosporin A 1:500. However, 100 µM THC reduced TNF-α, MMP-9, and ICAM-1 mRNA expression slightly more than 100 µM THDC.

Due to the anti-inflammatory effect of 1 µM curcumin, 10 µM curcumin, 100 µM curcumin, 100 µM BDC, 100 µM THC, 1 µM THDC, and 100 µM THDC, which were similar to cyclosporin A 1:500, these agents could be used as a treatment of DED. Most of these agents had better cell viability than cyclosporin A 1:500 except 100 µM curcumin and 100 µM BDC. As a result, 1 µM curcumin, 10 µM curcumin, 100 µM THC, 1 µM THDC, and 100 µM THDC needs to be studied further as a treatment for DED.

This study acknowledges certain limitations. We did not investigate the solubility, stability, and absorbability of curcumin analogs. Future bioavailability studies are recommended before their application in eye drop form for patients.

In conclusion, 1 and 10 µM curcumin, 100 µM THC, and 1 µM and 100 µM THDC can suppress TNF-α, IL-1β, IL-17 A, IL-6, MMP-9, and ICAM-1 mRNA expression in hyperosmotic stage-induced primary human corneal limbal epithelial cells as effectively as cyclosporin A 1:500 but with higher cell viability. Moreover, 100 µM curcumin and 100 µM BDC can suppress those inflammatory cytokines; however, the resulting cell viability is not significantly different from cyclosporin A 1:500. Aside from yellow curcumin and BDC, colorless 100 µM THC and 1 and 100 µM THDC have potential as novel eye drop treatments.

### Electronic supplementary material

Below is the link to the electronic supplementary material.

Supplementary Material 1

## Data Availability

The datasets generated/analyzed during the current study are available from the corresponding author upon reasonable request.

## Abbreviations

BDC: Bisdemethoxycurcumin
THC: Tetrahydrocurcumin
THDC: Tetrahydrodemethoxycurcumin
THBDC: Tetrahydrobisdemethoxycurcumin
ANOVA: Analysis of Variance
TNF: Tumor Necrosis Factor
MMP: Matrix Metalloproteinase
ICAM: Intercellular Adhesion Molecule
DED: Dry Eye Disease
TLC: Thin Layer Chromatography
DMEM: Dulbecco’s Modified Eagle Medium
PBS: Phosphate-Buffered Saline
EDTA: Ethylenediaminetetraacetic Acid
GAPDH: Glyceraldehyde-3-Phosphate Dehydrogenase
LFA: Lymphocyte Function-associated Antigen

## References

[1] Epstein J, Sanderson IR, Macdonald TT (2010). Curcumin as a therapeutic agent: the evidence from in vitro, animal and human studies. Br J Nutr.

[2] Nebbioso M, Franzone F, Greco A, Gharbiya M, Bonfiglio V, Polimeni A (2021). Recent advances and disputes about Curcumin in Retinal diseases. Clin Ophthalmol.

[3] Allegrini D, Raimondi R, Borgia A et al. Curcumin in Retinal diseases: a Comprehensive Review from Bench to Bedside. Int J Mol Sci. 2022;23. 10.3390/ijms23073557 PMC8998602 35408920

[4] Radkar P, Lakshmanan PS, Mary JJ, Chaudhary S, Durairaj SK (2021). A novel multi-ingredient supplement reduces inflammation of the Eye and improves production and quality of tears in humans. Ophthalmol Ther.

[5] Trivedi MK, Panda P, Sethi KK, Gangwar M, Mondal SC, Jana S (2020). Solid and liquid state characterization of tetrahydrocurcumin using XRPD, FT-IR, DSC, TGA, LC-MS, GC-MS, and NMR and its biological activities. J Pharm Anal.

[6] Vyas A, Dandawate P, Padhye S, Ahmad A, Sarkar F (2013). Perspectives on new synthetic curcumin analogs and their potential anticancer properties. Curr Pharm Des.

[7] Craig JP, Nichols KK, Akpek EK (2017). TFOS DEWS II definition and classification report. Ocul Surf.

[8] Jones L, Downie LE, Korb D (2017). TFOS DEWS II Management and Therapy Report. Ocul Surf.

[9] Nebbioso M, Fameli V, Gharbiya M, Sacchetti M, Zicari AM, Lambiase A (2016). Investigational drugs in dry eye disease. Expert Opin Investig Drugs.

[10] Changtam C, de Koning HP, Ibrahim H, Sajid MS, Gould MK, Suksamrarn A (2010). Curcuminoid analogs with potent activity against Trypanosoma and Leishmania species. Eur J Med Chem.

[11] Tocharus J, Jamsuwan S, Tocharus C, Changtam C, Suksamrarn A (2012). Curcuminoid analogs inhibit nitric oxide production from LPS-activated microglial cells. J Nat Med.

[12] Ohtsu H, Xiao Z, Ishida J (2002). Antitumor agents. 217. Curcumin analogues as novel androgen receptor antagonists with potential as anti-prostate cancer agents. J Med Chem.

[13] Lee SL, Huang WJ, Lin WW, Lee SS, Chen CH (2005). Preparation and anti-inflammatory activities of diarylheptanoid and diarylheptylamine analogs. Bioorg Med Chem.

[14] Portes E, Gardrat C, Castellan A (2007). A comparative study on the antioxidant properties of tetrahydrocurcuminoids and curcuminoids. Tetrahedron.

[15] Chen M, Hu DN, Pan Z, Lu CW, Xue CY, Aass I (2010). Curcumin protects against hyperosmoticity-induced IL-1beta elevation in human corneal epithelial cell via MAPK pathways. Exp Eye Res.

[16] Bron AJ, de Paiva CS, Chauhan SK (2017). TFOS DEWS II pathophysiology report. Ocul Surf.

[17] Roda M, Corazza I, Bacchi Reggiani ML et al. Dry Eye Disease and tear cytokine Levels-A Meta-analysis. Int J Mol Sci. 2020;21. 10.3390/ijms21093111 PMC7246678 32354090

[18] Schultz C (2014). Safety and efficacy of cyclosporine in the treatment of chronic dry eye. Ophthalmol Eye Dis.

[19] Haber SL, Benson V, Buckway CJ, Gonzales JM, Romanet D, Scholes B (2019). Lifitegrast: a novel drug for patients with dry eye disease. Ther Adv Ophthalmol.

[20] Liu XF, Hao JL, Xie T (2017). Curcumin, a potential therapeutic candidate for Anterior Segment Eye diseases: a review. Front Pharmacol.

[21] Zhang ZB, Luo DD, Xie JH (2018). Curcumin’s metabolites, Tetrahydrocurcumin and Octahydrocurcumin, Possess Superior anti-inflammatory effects in vivo through suppression of TAK1-NF-κB pathway. Front Pharmacol.

[22] Yoysungnoen B, Bhattarakosol P, Patumraj S, Changtam C (2015). Effects of tetrahydrocurcumin on hypoxia-inducible factor-1α and vascular endothelial growth factor expression in cervical cancer cell-induced angiogenesis in nude mice. Biomed Res Int.

[23] Chen BL, Chen YQ, Ma BH (2018). Tetrahydrocurcumin, a major metabolite of curcumin, ameliorates allergic airway inflammation by attenuating Th2 response and suppressing the IL-4Rα-Jak1-STAT6 and Jagged1/Jagged2 -Notch1/Notch2 pathways in asthmatic mice. Clin Exp Allergy.

[24] Pan Y, Zhang Y, Yuan J (2020). Tetrahydrocurcumin mitigates acute hypobaric hypoxia-induced cerebral oedema and inflammation through the NF-κB/VEGF/MMP-9 pathway. Phytother Res.

[25] Park S, Lee LR, Seo JH, Kang S (2016). Curcumin and tetrahydrocurcumin both prevent osteoarthritis symptoms and decrease the expressions of pro-inflammatory cytokines in estrogen-deficient rats. Genes Nutr.

[26] Kim JE, Kim HR, Kim JC (2021). Tetrahydrocurcumin ameliorates skin inflammation by modulating Autophagy in High-Fat Diet-Induced obese mice. Biomed Res Int.

[27] Stanić Z (2017). Curcumin, a compound from natural sources, a true scientific challenge - A review. Plant Foods Hum Nutr.

[28] Granata G, Paterniti I, Geraci C (2017). Potential Eye Drop based on a Calix[4]arene Nanoassembly for Curcumin Delivery: enhanced Drug Solubility, Stability, and anti-inflammatory effect. Mol Pharm.

[29] Pandey A, Chaturvedi M, Mishra S, Kumar P, Somvanshi P, Chaturvedi R (2020). Reductive metabolites of curcumin and their therapeutic effects. Heliyon.

[30] Kao YW, Hsu SK, Chen JY et al. Curcumin Metabolite Tetrahydrocurcumin in the treatment of Eye diseases. Int J Mol Sci. 2020;22. 10.3390/ijms22010212 PMC7795090 33379248

