# Supplementary material for: Anti-inflammatory effect of curcuminoids and their analogs in hyperosmotic human corneal limbus epithelial cells
Source: BMC Complement Med Ther. 2024 Apr 23;24:172. doi: 10.1186/s12906-024-04448-8 (PMC11040938; doi:10.1186/s12906-024-04448-8)
Supplement: Supplementary file 1 — Supplementary Material 1 [file 12906_2024_4448_MOESM1_ESM.docx]

**Table S1.** Primers used in quantitative reverse transcription polymerase chain reaction.

| Gene | Primer sequences | Reference |
| --- | --- | --- |
| TNF-α | Forward: 5′-CCC-AGG-CAG-TCA-GAT-CAT-CTT-3′  Reverse: 5′-AGC-TGC-CCC-TCA-GCT-TGA-3′ | [Zhang Q, et al.]^1^ |
| IL-1β | Forward: 5′-AAC-CTC-TTC-GAG-GCA-CAA-G-3′  Reverse: 5′-GTT-TAG-GGC-CAT-CAG-CTT-CA-3′ | [Zhang Q, et al.]^1^ |
| IL-6 | Forward: 5′-GGT-ACA-TCC-TCG-ACG-GCA-TCT-3′  Reverse: 5′-GTG-CTC-TTT-GCT-GCT-TTC-TCA-CAC-3′ | [Lin H, et al.]^2^ |
| IL-17A | Forward: 5′-CCC-CTA-GAC-TCA-GGC-TTC-CT-3′  Reverse: 5′-TCA-GCT-CCT-TTC-TGG-GTT-GT-3′ | [Kim SR, et al.]^3^ |
| MMP-9 | Forward: 5′-TGA-CAG-CGA-CAA-GAA-GTG-3′  Reverse: 5′-CAG-TGA-AGC-GGT-ACA-TAG-G-3′ | [Shan B, et al.]^4^ |
| ICAM-1 | Forward: 5′-ATG-CCC-AGA-CAT-CTG-TGT-CC-3′  Reverse: 5′-GGG-GTC-TCT-ATG-CCC-AAC-AA-3′ | [Gao H, et al.]^5^ |
| GAPDH | Forward: 5′-CAT-CAC-CAT-CTT-CCA-GGA-CG-3′  Reverse: 5′-GAG-GGG-CCA-TCC-ACA-GTC-TTC-3′ | [Tanaka T, et al.]^6^ |

TNF-α = tumor necrosis factor-α, IL-1β = interleukin-1β, IL-6 = interleukin-6, IL-17A = interleukin-17A, MMP-9 = matrix metallopeptidase 9, ICAM-1 = intercellular adhesion molecule 1, and GAPDH = glyceraldehyde-3-phosphate dehydrogenase.

**Table S2. Viability of primary human corneal limbal epithelial cells after exposure to each substance**

| Substances | Mean | SD | Lower 95% CI | Upper  95% CI |
| --- | --- | --- | --- | --- |
| Control | 100.00 | 3.45 | 91.43 | 108.60 |
| 90mM NaCl | 93.32 | 2.31 | 87.58 | 99.06 |
| 2mM H2O2 | 16.81 | 0.60 | 15.32 | 18.29 |
| Cyclosporin A 1:500 | 78.97 | 5.32 | 65.77 | 92.18 |
| 1 uM curcumin | 120.10 | 23.02 | 62.88 | 177.20 |
| 10 uM curcumin | 122.90 | 14.97 | 85.70 | 160.10 |
| 100 uM curcumin | 97.84 | 11.83 | 68.45 | 127.20 |
| 1 uM demethoxycurcumin | 123.20 | 6.84 | 106.20 | 140.20 |
| 10 uM demethoxycurcumin | 127.80 | 20.94 | 75.82 | 179.80 |
| 100 uM demethoxycurcumin | 119.30 | 14.13 | 84.21 | 154.40 |
| 1 uM bisdemethoxycurcumin | 131.00 | 8.00 | 111.10 | 150.90 |
| 10 uM bisdemethoxycurcumin | 122.90 | 14.07 | 87.96 | 157.80 |
| 100 uM bisdemethoxycurcumin | 90.95 | 5.41 | 77.53 | 104.40 |
| 1 uM tetrahydrocurcumin | 132.20 | 2.78 | 125.30 | 139.10 |
| 10 uM tetrahydrocurcumin | 127.00 | 5.75 | 112.70 | 141.30 |
| 100 uM tetrahydrocurcumin | 116.80 | 0.35 | 115.90 | 117.70 |
| 1 uM tetrahydrodemethoxycurcumin | 114.30 | 8.47 | 93.29 | 135.40 |
| 10 uM tetrahydrodemethoxycurcumin | 126.00 | 8.59 | 104.60 | 147.30 |
| 100 uM tetrahydrodemethoxycurcumin | 118.00 | 15.57 | 79.30 | 156.70 |
| 1 uM tetrahydrobisdemethoxycurcumin | 132.30 | 8.87 | 110.20 | 154.30 |
| 10 uM tetrahydrobisdemethoxycurcumin | 124.80 | 5.54 | 111.10 | 138.60 |
| 100 uM tetrahydrobisdemethoxycurcumin | 129.60 | 7.02 | 112.10 | 147.00 |

SD= standard deviation, CI= confidence interval

**Table S3. TNF-**$\boldsymbol{\alpha}$ **expression of stimulated primary human corneal limbus epithelial cells after receiving each substance**

| Substances | Mean | SD | Lower  95% CI | Upper  95% CI |
| --- | --- | --- | --- | --- |
| Control | 1.02 | 0.23 | 0.44 | 1.59 |
| 90mM NaCl 6 h | 21.67 | 3.65 | 12.61 | 30.72 |
| cyclosporin A 1:500 | 0.59 | 0.32 | -0.21 | 1.39 |
| 1uM CCM | 3.84 | 1.36 | 0.45 | 7.23 |
| 10uM CCM | 1.93 | 0.90 | -0.32 | 4.18 |
| 100uM CCM | 3.30 | 1.07 | 0.64 | 5.95 |
| 1uM DC | 7.36 | 3.49 | -1.32 | 16.04 |
| 10uM DC | 5.63 | 1.01 | 3.13 | 8.13 |
| 100uM DC | 10.86 | 1.82 | 6.35 | 15.37 |
| 1uM BDC | 9.27 | 4.31 | -1.43 | 19.96 |
| 10uM BDC | 10.03 | 1.38 | 6.60 | 13.45 |
| 100uM BDC | 5.63 | 0.05 | 5.50 | 5.76 |
| 1uM THC | 9.40 | 2.66 | 2.80 | 15.99 |
| 10uM THC | 9.05 | 0.77 | 7.15 | 10.96 |
| 100uM THC | 7.04 | 4.18 | -3.35 | 17.43 |
| 1uM THDC | 3.02 | 0.44 | 1.92 | 4.11 |
| 10uM THDC | 6.63 | 1.53 | 2.82 | 10.44 |
| 100uM THDC | 9.47 | 7.41 | -8.92 | 27.87 |
| 1uM THBDC | 9.16 | 5.31 | -4.04 | 22.36 |
| 10uM THBDC | 7.76 | 4.25 | -2.79 | 18.32 |
| 100uM THBDC | 8.62 | 3.12 | 0.87 | 16.38 |

SD= standard deviation, CI= confidence interval

**Table S4. IL-6 expression of stimulated primary human corneal limbus epithelial cells after receiving each substance**

| Substances | Mean | SD | Lower  95% CI | Upper  95% CI |
| --- | --- | --- | --- | --- |
| Control | 1.02 | 0.27 | 0.34 | 1.70 |
| 90mM NaCl 6 h | 28.85 | 2.48 | 22.68 | 35.02 |
| cyclosporin A 1:500 | 0.70 | 0.30 | -0.03 | 1.44 |
| 1uM CCM | 5.55 | 0.35 | 4.69 | 6.41 |
| 10uM CCM | 1.27 | 0.64 | -0.31 | 2.85 |
| 100uM CCM | 2.68 | 0.50 | 1.45 | 3.91 |
| 1uM DC | 13.97 | 6.56 | -2.32 | 30.26 |
| 10uM DC | 9.66 | 1.10 | 6.92 | 12.41 |
| 100uM DC | 3.39 | 0.74 | 1.56 | 5.22 |
| 1uM BDC | 11.13 | 4.93 | -1.13 | 23.38 |
| 10uM BDC | 0.18 | 0.10 | -0.05 | 0.42 |
| 100uM BDC | 2.07 | 0.78 | 0.12 | 4.01 |
| 1uM THC | 13.36 | 3.80 | 3.93 | 22.80 |
| 10uM THC | 9.35 | 2.19 | 3.90 | 14.80 |
| 100uM THC | 5.16 | 2.88 | -2.00 | 12.33 |
| 1uM THDC | 7.16 | 0.80 | 5.16 | 9.15 |
| 10uM THDC | 12.80 | 3.80 | 3.37 | 22.23 |
| 100uM THDC | 5.41 | 4.31 | -5.30 | 16.11 |
| 1uM THBDC | 13.06 | 6.17 | -2.27 | 28.38 |
| 10uM THBDC | 13.77 | 6.91 | -3.40 | 30.94 |
| 100uM THBDC | 6.91 | 3.06 | -0.69 | 14.52 |

SD= standard deviation, CI= confidence interval

**Table S5. IL-1**$\boldsymbol{\beta}$ **expression of stimulated primary human corneal limbus epithelial cells after receiving each substance**

| Substances | Mean | SD | Lower  95% CI | Upper  95% CI |
| --- | --- | --- | --- | --- |
| Control | 1.05 | 0.38 | 0.10 | 2.00 |
| 90mM NaCl 6 h | 4.32 | 0.68 | 2.62 | 6.02 |
| cyclosporin A 1:500 | 0.22 | 0.09 | 0.003 | 0.44 |
| 1uM CCM | 0.74 | 0.03 | 0.67 | 0.80 |
| 10uM CCM | 0.35 | 0.17 | -0.07 | 0.77 |
| 100uM CCM | 0.50 | 0.05 | 0.38 | 0.62 |
| 1uM DC | 1.75 | 0.94 | -0.59 | 4.09 |
| 10uM DC | 2.34 | 0.23 | 1.78 | 2.90 |
| 100uM DC | 1.72 | 0.36 | 0.82 | 2.62 |
| 1uM BDC | 2.56 | 0.36 | 1.67 | 3.46 |
| 10uM BDC | 0.001 | 0 | 0.01 | 0.01 |
| 100uM BDC | 0.84 | 0.15 | 0.46 | 1.22 |
| 1uM THC | 2.18 | 0.42 | 1.13 | 3.22 |
| 10uM THC | 2.46 | 0.56 | 1.07 | 3.86 |
| 100uM THC | 0.82 | 0.43 | -0.26 | 1.90 |
| 1uM THDC | 0.89 | 0.06 | 0.74 | 1.03 |
| 10uM THDC | 2.40 | 0.27 | 1.73 | 3.08 |
| 100uM THDC | 1.39 | 1.26 | -1.74 | 4.51 |
| 1uM THBDC | 1.91 | 0.95 | -0.46 | 4.28 |
| 10uM THBDC | 1.78 | 0.86 | -0.36 | 3.93 |
| 100uM THBDC | 1.07 | 0.71 | -0.70 | 2.84 |

SD= standard deviation, CI= confidence interval

**Table S6. IL-17A expression of stimulated primary human corneal limbus epithelial cells after receiving each substance**

| Substances | Mean | SD | Lower  95% CI | Upper  95% CI |
| --- | --- | --- | --- | --- |
| Control | 1.03 | 0.29 | 0.32 | 1.74 |
| 90mM NaCl 6 h | 59.64 | 19.31 | 11.68 | 107.60 |
| cyclosporin A 1:500 | 2.08 | 0.91 | -0.18 | 4.35 |
| 1uM CCM | 1.90 | 0.82 | -0.13 | 3.93 |
| 10uM CCM | 0.76 | 0.36 | -0.13 | 1.66 |
| 100uM CCM | 11.97 | 2.18 | 6.57 | 17.38 |
| 1uM DC | 8.60 | 4.22 | -1.88 | 19.08 |
| 10uM DC | 2.23 | 0.43 | 1.16 | 3.30 |
| 100uM DC | 1.67 | 0.47 | 0.51 | 2.82 |
| 1uM BDC | 9.84 | 2.24 | 4.28 | 15.39 |
| 10uM BDC | 16.77 | 0.88 | 14.59 | 18.96 |
| 100uM BDC | 12.23 | 2.32 | 6.46 | 17.99 |
| 1uM THC | 16.43 | 4.61 | 4.98 | 27.89 |
| 10uM THC | 4.95 | 1.29 | 1.73 | 8.16 |
| 100uM THC | 2.72 | 2.06 | -2.40 | 7.83 |
| 1uM THDC | 5.24 | 0.58 | 3.80 | 6.69 |
| 10uM THDC | 13.19 | 1.65 | 9.09 | 17.30 |
| 100uM THDC | 2.37 | 2.00 | -2.61 | 7.34 |
| 1uM THBDC | 4.62 | 2.09 | -0.57 | 9.81 |
| 10uM THBDC | 15.16 | 5.76 | 0.86 | 29.45 |
| 100uM THBDC | 15.04 | 4.07 | 4.92 | 25.15 |

SD= standard deviation, CI= confidence interval

**Table S7. ICAM-1 expression of stimulated primary human corneal limbus epithelial cells after receiving each substance**

| Substances | Mean | SD | Lower  95% CI | Upper  95% CI |
| --- | --- | --- | --- | --- |
| Control | 1.03 | 0.31 | 0.26 | 1.81 |
| 90mM NaCl 6 h | 17.97 | 4.48 | 6.84 | 29.10 |
| cyclosporin A 1:500 | 0.72 | 0.25 | 0.10 | 1.35 |
| 1uM CCM | 3.27 | 0.04 | 3.17 | 3.37 |
| 10uM CCM | 1.37 | 0.61 | -0.15 | 2.88 |
| 100uM CCM | 2.19 | 0.37 | 1.27 | 3.12 |
| 1uM DC | 5.23 | 2.56 | -1.13 | 11.58 |
| 10uM DC | 7.07 | 1.21 | 4.08 | 10.07 |
| 100uM DC | 5.37 | 0.36 | 4.48 | 6.27 |
| 1uM BDC | 9.29 | 2.00 | 4.33 | 14.25 |
| 10uM BDC | 0.09 | 0.02 | 0.06 | 0.13 |
| 100uM BDC | 2.60 | 0.50 | 1.37 | 3.83 |
| 1uM THC | 7.71 | 1.75 | 3.38 | 12.05 |
| 10uM THC | 4.43 | 0.88 | 2.25 | 6.61 |
| 100uM THC | 4.92 | 2.38 | -1.00 | 10.84 |
| 1uM THDC | 4.40 | 0.30 | 3.66 | 5.14 |
| 10uM THDC | 8.68 | 1.94 | 3.87 | 13.49 |
| 100uM THDC | 7.90 | 7.63 | -11.05 | 26.85 |
| 1uM THBDC | 8.40 | 3.38 | 0.01 | 16.79 |
| 10uM THBDC | 8.78 | 4.39 | -2.12 | 19.68 |
| 100uM THBDC | 5.88 | 2.27 | 0.25 | 11.52 |

SD= standard deviation, CI= confidence interval

**Table S8. MMP-9 expression of stimulated primary human corneal limbus epithelial cells after receiving each substance**

| Substances | Mean | SD | Lower  95% CI | Upper  95% CI |
| --- | --- | --- | --- | --- |
| Control | 1.04 | 0.35 | 0.17 | 1.91 |
| 90mM NaCl 6 h | 62.10 | 11.21 | 34.25 | 89.95 |
| cyclosporin A 1:500 | 1.57 | 0.64 | -0.03 | 3.17 |
| 1uM CCM | 9.52 | 0.24 | 8.93 | 10.11 |
| 10uM CCM | 2.00 | 0.94 | -0.33 | 4.33 |
| 100uM CCM | 0.20 | 0.03 | 0.12 | 0.28 |
| 1uM DC | 20.02 | 9.50 | -3.59 | 43.62 |
| 10uM DC | 21.92 | 2.98 | 14.51 | 29.33 |
| 100uM DC | 4.78 | 0.74 | 2.95 | 6.61 |
| 1uM BDC | 33.30 | 3.66 | 24.21 | 42.39 |
| 10uM BDC | 0.003 | 0.006 | -0.01 | 0.02 |
| 100uM BDC | 0.43 | 0.13 | 0.11 | 0.74 |
| 1uM THC | 23.72 | 7.08 | 6.15 | 41.29 |
| 10uM THC | 23.06 | 4.46 | 11.97 | 34.15 |
| 100uM THC | 13.98 | 6.87 | -3.07 | 31.04 |
| 1uM THDC | 7.85 | 1.05 | 5.25 | 10.45 |
| 10uM THDC | 29.51 | 4.48 | 18.38 | 40.63 |
| 100uM THDC | 21.72 | 19.33 | -26.30 | 69.74 |
| 1uM THBDC | 19.23 | 9.70 | -4.86 | 43.32 |
| 10uM THBDC | 24.99 | 10.79 | -1.81 | 51.80 |
| 100uM THBDC | 30.22 | 12.42 | -0.64 | 61.08 |

SD= standard deviation, CI= confidence interval

**Reference**

1. Zhang Q, Kandic I, Barfield JT, Kutryk MJ. Coculture with Late, but Not Early, Human Endothelial Progenitor Cells Up Regulates IL-1 β Expression in THP-1 Monocytic Cells in a Paracrine Manner. Stem Cells Int. 2013;2013:859643.

2. Lin H, Li N, He H, et al. AMPK Inhibits the Stimulatory Effects of TGF-β on Smad2/3 Activity, Cell Migration, and Epithelial-to-Mesenchymal Transition. Mol Pharmacol. 2015;88:1062-1071.

3. Kim SR, Kim HJ, Kim DI, et al. Blockade of Interplay between IL-17A and Endoplasmic Reticulum Stress Attenuates LPS-Induced Lung Injury. Theranostics. 2015;5:1343-1362.

4. Shan B, Zhuo Y, Chin D, Morris CA, Morris GF, Lasky JA. Cyclin-dependent kinase 9 is required for tumor necrosis factor-alpha-stimulated matrix metalloproteinase-9 expression in human lung adenocarcinoma cells. J Biol Chem. 2005;280:1103-1111.

5. Gao H, Liu L, Zhao Y, et al. Human IL-6, IL-17, IL-1β, and TNF-α differently regulate the expression of pro-inflammatory related genes, tissue factor, and swine leukocyte antigen class I in porcine aortic endothelial cells. Xenotransplantation. 2017;24.

6. Tanaka T, Obana M, Mohri T, et al. Interleukin-27 induces the endothelial differentiation in Sca-1+ cardiac resident stem cells. Cytokine. 2015;75:365-372.
